# Periungual squamous cell carcinoma in childhood: a case report

**DOI:** 10.1093/omcr/omae216

**Published:** 2025-03-28

**Authors:** Marysol Macedo-Pérez, Stefanie Arroyo-Camarena, Rodrigo Roldán-Marín, Sonia Toussaint-Caire, Froylan D Martínez-Sánchez

**Affiliations:** Department of Dermatology, Hospital General Dr. Manuel Gea Gonzalez, Calz. de Tlalpan 4800, Belisario Domínguez Secc 16, Tlalpan, Ciudad de Mexico 14080, Mexico; Clínica de Oncodermatología, Facultad de Medicina, Dr. Balmis 148, Doctores, Coyoacán, Ciudad de México 06720, Mexico; Clínica de Oncodermatología, Facultad de Medicina, Dr. Balmis 148, Doctores, Coyoacán, Ciudad de México 06720, Mexico; Department of Dermatopathology, Hospital General Dr. Manuel Gea Gonzalez, Calz. de Tlalpan 4800, Belisario Domínguez Secc 16, Tlalpan, Ciudad de Mexico 14080, Mexico; Department of Internal Medicine, Hospital General Dr. Manuel Gea Gonzalez, Calz. de Tlalpan 4800, Belisario Domínguez Secc 16, Tlalpan, Ciudad de Mexico 14080, Mexico; Facultad de Medicina, Universidad Nacional Autonoma de Mexico. Escolar 411A, Copilco Universidad, Coyoacán 04360 Ciudad de México

**Keywords:** squamous cell carcinoma, periungual, cancer

## Abstract

Bowen's disease (BD) is the second most common skin cancer worldwide, often linked to sun exposure, arsenic, and immunosuppression. Though rare in pediatrics, it is the third most common pediatric skin cancer, associated with conditions like albinism and immunosuppression. Recent years have seen an increase in Squamous Cell Carcinoma (SCC) cases among children, potentially related to immunosuppression and human papillomavirus (HPV) infection, particularly subtype 16. We present the case of an 11-year-old girl from Mexico City with a five-year history of a slowly growing, asymptomatic, dark-brown plaque on the periungual region of her right hand. Histopathological examination revealed Pigmented Squamous Cell Carcinoma in situ. The lesion was treated with excision, nail apparatus removal, and adjuvant 5% imiquimod cream, followed by skin grafting. This case underscores the importance of considering malignancy in pediatric patients, particularly with atypical presentations in acral sites, and highlights the diagnostic challenges of pigmented SCC.

## Introduction

Bowen's disease (BD), also known as squamous cell carcinoma (SCC) in situ, represents a significant concern in dermatology due to its prevalence and potential for malignancy. Globally, BD is recognized as the second most common form of skin cancer, often associated with risk factors such as prolonged sun exposure, arsenic exposure, radiotherapy, trauma, immunosuppressed states, and actinic keratosis [1, 2]. The primary environmental cause of cutaneous SCC is solar ultraviolet radiation (UVR), particularly in individuals with fair skin who have high sun sensitivity [1]. Immunosuppression also plays a crucial role, as evidenced by the high incidence of SCC in organ transplant recipients and individuals with chronic immunosuppressive conditions [1]. Although BD is rare in pediatric populations, it is considered the third most common skin cancer in children, frequently associated with genetic conditions such as albinism, epidermolysis bullosa, xeroderma pigmentosum, or immunosuppression [3, 4]. Recent studies have highlighted an increase in the incidence of SCC among children, potentially linked to immunosuppression and human papillomavirus (HPV) infection, particularly subtype 16, which is commonly found in BD [4–7]. Although uncommon, isolated case reports of subungual SCC in pediatric patients underscore the need for heightened awareness and early detection [6, 7].

## Case report

**Figure 1:**
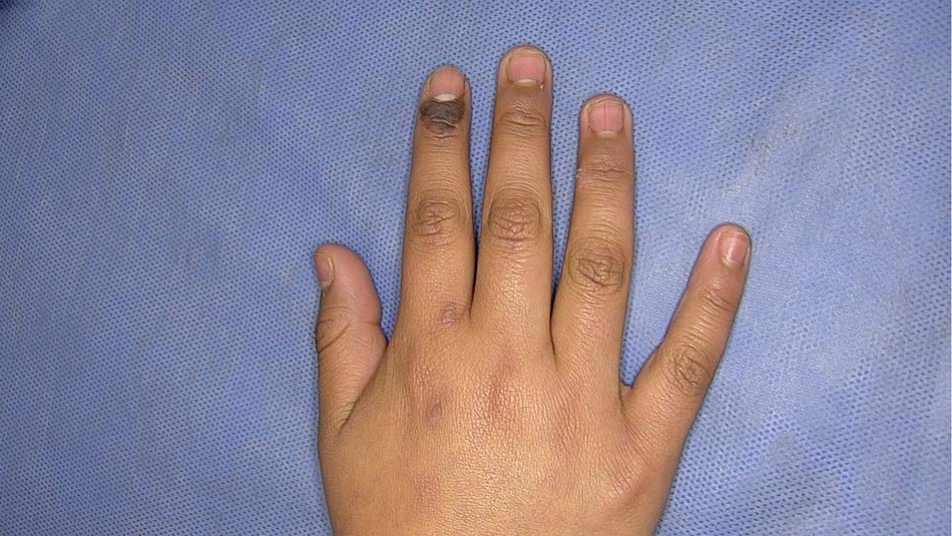
Clinical image of the 11-year-old patient's periungual plaque on the second finger of her right hand.

**Figure 2:**
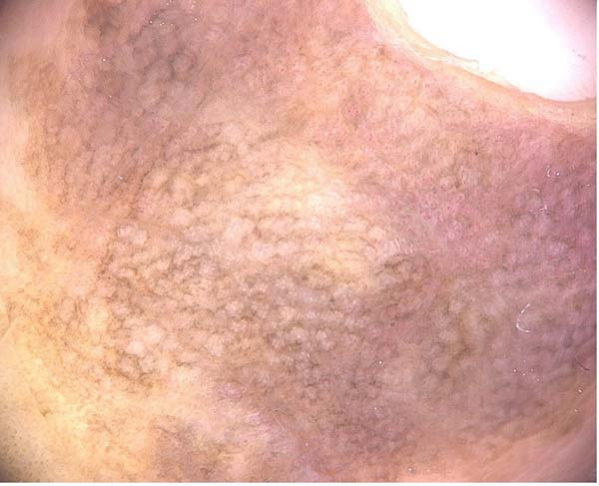
Dermoscopic image showing the pigment network with focal scattered dots.

**Figure 3:**
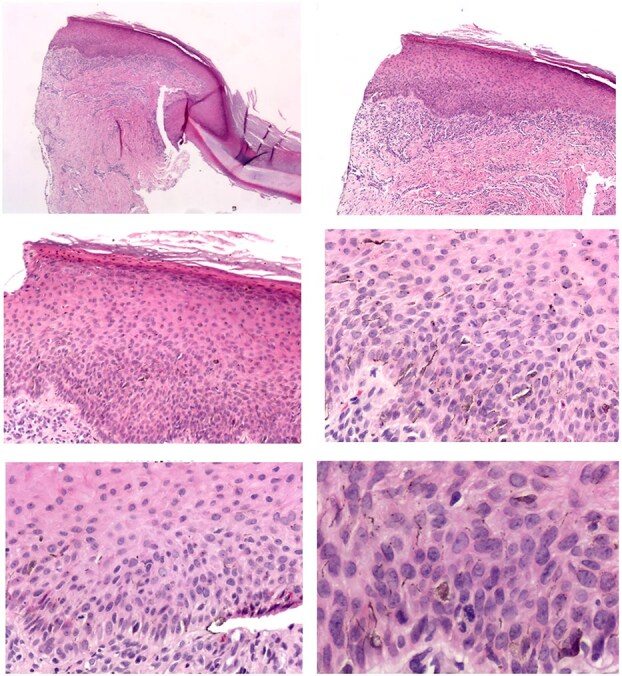
Biopsy description. The excisional biopsy of the nail apparatus, measuring approximately 1.7 × 1.7 cm, included the nail plate and surrounding acral skin. Histopathological examination revealed a hyperplastic epidermis with focal areas of confluent parakeratosis. The lesion showed acanthosis with elongation and widening of the rete ridges, composed of large keratinocytes with moderate cytological atypia. These cells exhibited abundant eosinophilic cytoplasm, pleomorphic hyperchromatic nuclei, and coarse chromatin. Suprabasal mitoses and individual cell necrosis were observed, with some keratinocytes containing melanin granules. An inflammatory infiltrate, primarily composed of lymphocytes and histiocytes, was noted in the perivascular regions beneath the lesion. The proximal portion of the acral skin near the nail fold displayed residual foci of pigmented squamous cell carcinoma in situ. In contrast, the nail matrix, bed, and hyponychium were free of tumor involvement.

We report the case of an 11-year-old girl from Mexico City with no significant family or personal medical history. The patient presented with a five-year history of a slowly growing, asymptomatic, dark-brown plaque in the periungual region of the second finger on her right hand (Fig. 1). A dermoscopic examination using polarized light (Fotofinder, 20×, HD Medicam 800) revealed a brown pigment network with focal scattered brown-gray dots (Fig. 2). An incisional biopsy was performed, and a histopathological examination demonstrated a hyperplastic epidermis with hyperkeratosis and foci of confluent parakeratosis. The biopsy also revealed acanthosis with elongated and widened epidermal rete ridges displaying full-thickness keratinocytic atypia. The keratinocytes were large, with abundant eosinophilic cytoplasm, pleomorphic and hyperchromatic nuclei, and prominent nucleoli. Suprabasal mitoses and individual cell necrosis were observed, with some atypical keratinocytes containing melanin granules within their cytoplasm (Fig. 3). The diagnosis was confirmed as Pigmented SCC in situ of acral skin. The patient underwent two peripheral skin margin excisions, removal of the nail apparatus, and treatment with 5% imiquimod cream as an adjuvant. The tissue defect was repaired with a skin graft, and there has been no lesion recurrence during a two-year follow-up period.

## Discussion

This case of Pigmented SCC in situ in an 11-year-old girl is particularly significant due to its atypical presentation and the challenges it poses in diagnosis. Although the patient did not present any clinical history of known predisposing factors, such as glucocorticoid use, immunosuppressive therapy (e.g. methotrexate, cyclosporine), or other treatments associated with immunosuppression, no specific genetic studies were performed to rule out hereditary risk factors. The subtle clinical appearance as an asymptomatic brownish plaque, absence of pre-existing dermatosis, early age of onset, and lack of typical risk factors, such as immunosuppression or genetic predispositions like albinism or xeroderma pigmentosum, contributed to the diagnostic complexity [1].

SCC is exceedingly rare in pediatric populations, making this case unusual [1, 2]. Typically, SCCs are more common in adults, particularly those with prolonged exposure to UV radiation or immunocompromised [1]. Chow et al. (2007) noted an increase in SCC cases in children, potentially linked to improved survival rates in children with other cancers and the associated long-term effects of treatments such as radiotherapy and chemotherapy [6].

HPV, particularly subtype 16, has been implicated in the pathogenesis of various SCCs, including those of the nail unit and periungual regions. In a study by Perruchoud et al. (2016), HPV DNA was detected in a significant number of Bowen's disease cases, highlighting the virus's role in these malignancies [2].

The location of the lesion in the periungual area added to the diagnostic challenge, as these sites are often overlooked in routine examinations [7]. Dermoscopy played a crucial role in identifying the pigmented network that guided the decision for a biopsy, ultimately leading to the correct diagnosis. The importance of thorough clinical evaluation and diagnostic tools like dermoscopy, particularly in atypical cases, cannot be overstated.

Senerchia et al. (2014) noted that the incidence of non-melanoma skin cancers in young adults has increased. While these cancers remain rare in children, they should be considered, especially in cases where lesions do not respond to conventional treatments or continue to grow [5]. The histopathological findings in this case, including keratinocytic atypia and melanin granules, were crucial for distinguishing Pigmented SCC in situ from other pigmented lesions such as nevi or melanoma [5].

In conclusion, this case underscores the need for heightened clinical suspicion and a proactive approach to diagnosing atypical pigmented pediatric lesions. Despite its rarity in this age group, SCC should be considered in the differential diagnosis, particularly in the absence of traditional risk factors. Early biopsy and appropriate diagnostic tools like dermoscopy ensure accurate diagnosis and timely management, potentially improving patient outcomes [5].

Subungual SCC represents a challenging diagnostic and therapeutic entity due to its rarity and the nonspecific nature of its clinical presentation [7, 8]. As Hinchcliff et al. (2019) highlighted, subungual SCC commonly manifests with symptoms such as nail deformity, hyperkeratosis, discoloration, and ulceration—signs that are easily mistaken for more benign conditions like fungal infections or trauma-related changes. This diagnostic ambiguity is particularly problematic, as subungual SCC can often lead to significant local invasion, including the involvement of the distal phalanx in a substantial number of cases [8].

The approach to subungual SCC requires a high degree of clinical suspicion, especially in cases where standard treatments fail to resolve the lesion within a typical timeframe [8]. Diagnostic imaging, particularly plain radiographs, is crucial in evaluating potential bone involvement, which occurs in 20% to 50% of cases [8]. The definitive diagnosis, however, hinges on histopathological examination following a biopsy. The characteristic features of SCC, such as keratin pearls, pleomorphic keratinocytes, and frequent mitoses, are essential for distinguishing it from other subungual pathologies [6–8].
